# Uremic Toxins and Vascular Dysfunction

**DOI:** 10.3390/toxins12060404

**Published:** 2020-06-18

**Authors:** Isabelle Six, Nadia Flissi, Gaëlle Lenglet, Loïc Louvet, Said Kamel, Marlène Gallet, Ziad A. Massy, Sophie Liabeuf

**Affiliations:** 1 UR 7517 UPJV, Pathophysiological Mechanisms and Consequences of Cardiovascular Calcifications (MP3CV), Picardie Jules Verne University, 80025 Amiens, France; nadia.flissi@etud.u-picardie.fr (N.F.); gaelle.lenglet@u-picardie.fr (G.L.); loic.louvet@u-picardie.fr (L.L.); said.kamel@u-picardie.fr (S.K.); marlene.gallet@u-picardie.fr (M.G.); liabeuf.sophie@chu-amiens.fr (S.L.); 2 Amiens-Picardie University Hospital, Human Biology Center, 80054 Amiens, France; 3 Service de Néphrologie et Dialyse, Assistance Publique—Hôpitaux de Paris (APHP), Hôpital Universitaire Ambroise Paré, 92100 Boulogne Billancourt, France; ziad.massy@aphp.fr; 4 INSERM U1018, Equipe 5, CESP (Centre de Recherche en Épidémiologie et Santé des Populations), Université Paris Saclay et Université Versailles Saint Quentin en Yvelines, 94800 Villejuif, France; 5 Pharmacology Department, Amiens University Hospital, 80025 Amiens, France

**Keywords:** chronic kidney disease, uremic toxins, vascular dysfunction

## Abstract

Vascular dysfunction is an essential element found in many cardiovascular pathologies and in pathologies that have a cardiovascular impact such as chronic kidney disease (CKD). Alteration of vasomotricity is due to an imbalance between the production of relaxing and contracting factors. In addition to becoming a determining factor in pathophysiological alterations, vascular dysfunction constitutes the first step in the development of atherosclerosis plaques or vascular calcifications. In patients with CKD, alteration of vasomotricity tends to emerge as being a new, less conventional, risk factor. CKD is characterized by the accumulation of uremic toxins (UTs) such as phosphate, para-cresyl sulfate, indoxyl sulfate, and FGF23 and, consequently, the deleterious role of UTs on vascular dysfunction has been explored. This accumulation of UTs is associated with systemic alterations including inflammation, oxidative stress, and the decrease of nitric oxide production. The present review proposes to summarize our current knowledge of the mechanisms by which UTs induce vascular dysfunction.

## 1. Introduction

The vascular wall is composed of three differentially structured layers from the periphery to the lumen of the vessel, i.e., the adventitia, the media composed mainly of smooth muscle cells, and the intima composed essentially of endothelial cells. Vasomotricity is the capacity of the vessel to contract or relax. To do this, the endothelium responds to stimuli and translates them into biological responses transmitted to the smooth muscle. We can distinguish vasomotricity that is due to the direct action of the smooth muscle and vasomotricity that is dependent on endothelium. The vasomotor responses of the smooth muscle are related to the activation of signaling pathways which result in variations in intracellular calcium concentrations [1]. The resulting cytosolic increase of calcium leads to vasoconstriction, while decreased calcium leads to vasorelaxation. The endothelium-dependent vasoconstriction is linked to the production of factors produced by endothelium (derivatives of arachidonic acid metabolism: endoperoxides, thromboxane A2, prostaglandin H2, endothelin, reactive oxygen species (ROS), and angiotensin II, AngII) resulting in a smooth muscle contraction. Endothelial cells are able to modulate vasomotricity through the production of relaxing factors such as prostacyclin (PGI2), nitric oxide (NO), and endothelium-derived hyperpolarizing factor (EDHF) [2]. Vascular function and its maintenance are essential elements in ensuring the health of the vessel.

Vascular dysfunction characterizes an imbalance between production of relaxing and contracting factors. Alternatively, vascular dysfunction can also be related to a decrease in the sensitivity of vascular smooth muscle cells (VSMCs) to relaxing factors produced by the endothelium or to an overproduction of ROS in the endothelial cells or the VSMCs.

Vascular dysfunction is an essential element found in many pathologies of cardiovascular origin or pathologies that have a cardiovascular impact such as chronic kidney disease (CKD). In addition to becoming a determining factor in pathophysiological courses, alteration of vascular function would constitute the developmental bed for the morphological alterations of the vessels characterized, in particular, by the presence of atheromatous plaques or vascular calcifications. In patients with CKD, cardiovascular disease is the leading cause of death and vascular dysfunction tends to emerge as being a new, less conventional risk factor [3].

CKD is characterized by the accumulation of uremic toxins (UTs) such as inorganic phosphate (Pi), para-cresyl sulfate (PCS), indoxyl sulfate (IS), and fibroblast growth factor 23 (FGF23). The deleterious role of UTs on vascular dysfunction has been explored. The accumulation of UTs has been reported to lead to inflammation [4,5], oxidative stress [6], anemia [7], ROS modulations [8], a decrease in NO production [9,10], as well as an increased production of asymmetric dimethylarginine (ADMA), an endogenous inhibitor of NO synthase [11].

This review proposes to summarize our current knowledge on the mechanisms by which UTs induce vascular dysfunction and to describe a few potential medical treatment options to lower the level of UTs and decrease their effects on vascular dysfunction.

## 2. The Vascular Function

**Figure 1:**
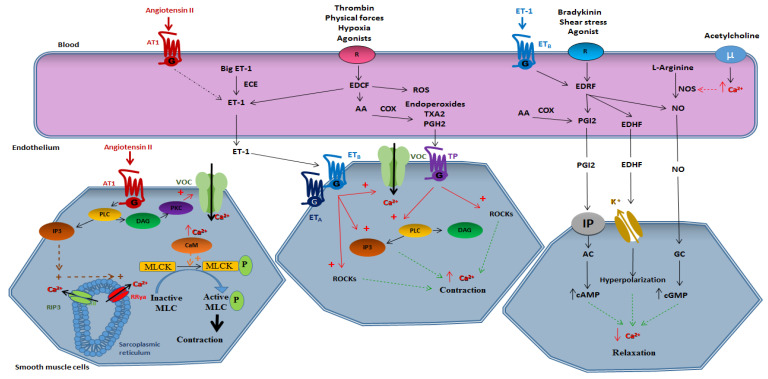
Vasoactive substances. AT1, angiotensin II receptor type I; ET-1, endothelin 1; ECE, endothelin converting enzyme; R, receptor; EDCF, endothelium-derived contracting factor; ROS, reactive oxygen species; AA, arachidonic acid, PGH2, prostaglandin H2; PGI2, prostacyclin; TXA2, thromboxan A2; COX, cyclooxygenase; EDRF, endothelium-derived relaxing factor; EDHF, endothelium-derived hyperpolarizing factor; NO, nitric oxide; NOS, NO synthase; µ, µ opioide receptor; PLC, phospholipase C; DAG, diacylglycerol; IP3, inositol trisphosphate; PKC, protein kinase C; RIP3, inositol trisphosphate receptor; RRya, ryanodin receptor; VOC, voltage operated channel; Ca^2+^, calcium; CaM, calmodulin; MLCK, myosin light chain kinase; MLC, myosin light chain; ETA/ETB, endothelin receptors A/B; TP, TP receptor; K^+^, potassium; IP, prostacyclin receptor; AC, adenylate cyclase; GC, guanylate cyclase; cAMP, cyclic adenosin monophosphate; cGMP, cyclic guanosine monophosphate.

Figure 1 provides a schematic view of the mechanism implicated in vasomotricity.

Vasomotricity is the ability of the vessel to contract and to relax in response to chemical stimuli such as hormones, growth factors, or cytokines and physical stimuli such as shear stress. We can distinguish vasomotricity related to the direct action on the smooth muscle and vasomotricity dependent on endothelium.

### 2.1. Smooth Muscle-Dependent Vasomotricity

The vasomotor responses of the smooth muscle are related to intracellular calcium concentration. Since calcium homeostasis is essential in the smooth muscle cell, this cell must be able to adjust calcium concentration very quickly.

#### 2.1.1. Vasoconstriction

A contraction can occur during the transient increase in intracellular calcium levels. However, a contraction is possible at low intracellular calcium concentrations due to a contractile sensitization phenomenon whose mechanisms are mainly related to the inhibition of the myosin light chain phosphatase (MLCP) activity and the phosphorylation of regulatory proteins [1].

Briefly, a contraction is induced through the opening of the membrane voltage operated channel (VOCs) of L type. It can also be done by activating G-protein-coupled receptors (GPCRs). Vasoactive peptides, such as angiotensin II (Ang II), bind to GPCRs, leading to the activation of phospholipase C (PLC), and thus the production of inositol trisphosphate (IP3) and diacylglycerol (DAG). The released IP3 stimulates the opening of the IP3 receptors (RIP3) and, subsequently, the opening of the ryanodine receptors (RRya), both are settled in the sarcoplasmic reticulum and their opening allows the massive release of calcium from the internal stores to the cytosol. Intracellular calcium elevation, as well as DAG activation of the protein kinase C (PKC), modulate calcium channels at the plasma membrane and the sodium/calcium exchanger NCX, and therefore, further calcium entry from the extracellular space to the cytosol. This phenomenon induces activation of calmodulin, which changes conformation and loses its inhibitory effect on actin, and this mechanism allows the interaction of calmodulin with the myosin light chain kinase (MLCK). The activated MLCK phosphorylates the myosin light chain (MLC) leading to the actin-myosin interaction, and thus contraction of the smooth muscle cell. Therefore, the contraction is dependent on the phosphorylation/dephosphorylation states of MLC. Any factors altering the phosphorylation state of MLC play a role in the contraction abilities of the smooth muscle cell regardless of intracellular calcium concentration.

#### 2.1.2. Vasorelaxation

Endothelium-independent relaxation is linked to a decrease of calcium concentrations at the intracellular level in VSMCs. A decline in the amount of intracellular calcium is linked to two mechanisms. On the one hand, the extrusion of calcium by membrane calcium ATPases pumps and sodium/calcium exchangers and, on the other hand, the uptake of calcium contained in the cytosol to internal stores mainly via the SERCA (sarco endoplasmic reticulum calcium ATPase) pumps [12]. A decrease in the amount of intracellular calcium leads to a decrease in the activity of MLCK. In addition, active MLCP is responsible for the dephosphorylation of MLC, and therefore vasorelaxation.

### 2.2. Endothelium-Dependent Vasomotricity

#### 2.2.1. Vasoconstriction

Endothelium-dependent vasoconstriction involves all mechanisms of action in which the factors produced by the endothelium induce a contraction of the smooth muscle. This vasoconstriction is activated by substances such as thrombin, pharmacological agents, physical forces, and hypoxia. A significant number of endothelium-derived contracting factors have been described but some factors remain to be identified.

The contracting factors produced by endothelium are called endothelium-derived contracting factors (EDCF). They are represented by the following derivatives of arachidonic acid metabolism: endoperoxides, thromboxane A2 (TXA2), prostaglandin H2 (PGH2), as well as endothelin and ROS [13]. In addition to the synthesis of contracting factors, the endothelium participates in activation or storage of vasoconstrictive substances. Thus, the angiotensin converting enzyme, which is present in endothelial cells, generates a powerful vasoconstrictor by converting angiotensin I into Ang II.

##### Derivatives of Arachidonic Acid

Arachidonic acid derivatives are contracting elements called endoperoxides that are the source of prostaglandins, i.e., prostaglandin E2 (PGE2), prostaglandin F2 (PGF2) and PGI2, and TXA2. All endoperoxides are able to induce endothelium-dependent vasoconstriction. At the vascular level, the PGH2 and TXA2, released by the endothelium, attach to the TP receptor (thromboxane receptor) expressed in the smooth muscle cells. The activation of this GPCR leads to the activation of PLC, the production of IP3 and DAG and, ultimately, to an increase in intracellular calcium concentration. These elements are also capable of inducing contraction by activating Rho-associated protein kinase (ROCK).

##### Endothelin 1

Under physiological conditions, endothelin is mainly produced at the endothelium level in the form of ET-1 [14]. Type A endothelin receptors (ETA) are found only in the smooth muscle and type B endothelin receptors (ETB) are present in the endothelium and smooth muscle. Endothelin has a direct effect on smooth muscle cells which causes the contraction of the smooth muscle and also has an indirect effect, via NO, causing relaxation of the smooth muscle. The contracting effect of endothelin outweighs the relaxing effect, which remains very small and is almost negligible [15].

At the level of the smooth muscle, ET-1 induces contraction in the following two ways: (1) by attaching to its ETA and ETB RCPG causing activation of VOCs, the production of IP3, and therefore the intracellular increase in calcium and (2) by activating the ROCK’s pathway.

##### Reactive Oxygen Species

Oxidative stress is characterized by an increase in ROS production. In response to different stimuli, endothelial cells produce hydrogen peroxide (H_2_O_2_) and superoxide anion O_2_**^.^-**. ROS acts by inducing vasoconstriction or by decreasing NO-induced vasorelaxation [16,17,18]. The precursor of ROS, O_2_**^.^-** is produced at the endothelial level by reducing the molecular oxygen O_2_ by type 1 cyclooxygenase (COX-1). Its action on contraction is specific to certain species and vascular beds and is characterized by a decrease in the effects of NO [19,20] and a direct action on guanylate cyclase, thus, decreasing its effects [16]. Hydrogen peroxide (H_2_O_2_) is obtained by reducing the superoxide anion O_2_**^.^-** by superoxide dismutase (SOD) and H_2_O_2_ induces contraction by activating COXs and inducing the production of TXA2 [21,22,23].

Lastly, the radical hydroxyl OH**^.^** is produced by Fenton’s reaction from hydrogen peroxide and also induces the contraction of smooth muscle cells via an increase in intracellular calcium level in the smooth muscle cell [24].

##### Angiotensin II

Ang II is a powerful contracting factor. Within the renin angiotensin system, angiotensinogen is produced mainly at the liver level and it is transformed by renin into angiotensin I which itself is transformed by the angiotensin converting enzyme to Ang II. At the endothelial level, Ang II attaches to the angiotensin type 1 receptors (AT1), thus, allowing the release of ET-1. Endothelin, by binding to its receptor at the smooth muscle cell level, activates the PLC, allows the production of IP3, induces the release of calcium, and also allows the activation of ROCK leading to smooth muscle contraction.

#### 2.2.2. Vasorelaxation

Endothelial cells are able to modulate vasomotricity through the production of relaxing factors. There are essentially three vasodilatory molecules produced by endothelium, i.e., PGI2, NO, and EDHF.

##### Prostacycline PGI2

PGI2 was the first vasoactive substance derived from endothelium discovered in the late of 1970s [25]. It is produced at the endothelial cell level from arachidonic acid under the action of COXs. PGI2 diffuses from the endothelial cell to the smooth muscle cell where it binds to its IP receptor (prostaglandin I2 receptor). The binding of PGI2 to its receptor results in the activation of adenylate cyclase which in turn results in an increase in the production of cyclic adenosine monophosphate (cAMP) which leads to relaxation.

##### Nitric Oxide (NO)

As early as 1980, Furchgott and Zawadski observed that the relaxation of isolated arteries subjected to the action of acetylcholine depended on endothelium. They deduced that a factor, they called EDRF (endothelium-derived relaxing factor), secreted by endothelial cells caused the relaxation of adjacent smooth muscle cells [26]. Many substances such as acetylcholine, serotonin, and bradykinin have induced the release of NO by acting on specific membrane receptors. The most important physiological stimulus causing NO production is the friction of the blood on the endothelium which varies with flow and flow conditions [16]. PGI2 and NO act synergistically by two distinct intracellular signal transduction mechanisms, i.e., the cAMP pathway for PGI2 and the cyclic monophosphate guanosine (cGMP) for NO.

NO is an original intercellular communication agent. It does not have a specific membrane receptor and acts without triggering a cascade of second messengers. NO diffuses from the emitting cell (endothelial cell) to the receiving cell (VSMC) where it acts directly on intracellular molecular targets. NO is produced from the terminal guanido nitrogen atom of L-arginine by NO synthase (NOS). It activates guanylate cyclase present in smooth muscle cells which results in the synthesis of cGMP and vessel relaxation. Many NOS isoforms have been identified. They can be constitutive and require the presence of calcium and calmodulin which is the case for endothelial (eNOS or type III) and neural (nNOS or type I) isoforms. These constitutive isoforms have a fast, short-lived action (a few minutes) and produce small amounts of NO (nM). Other isoforms can be inducible (iNOS or type II) and do not require the presence of calcium. They are found in many cell types (endothelial cells, smooth muscle cells, etc.) after stimulation by endotoxin, TNF, or other cytokines [27,28,29]. The induction of these isoforms results in a significant production (M) during a prolonged period (a few days) of NO.

The activation of eNOS results from a dissociation of this complex and is regulated by intracellular calcium, which when it increases, leads to the binding of calcium to the calmodulin calcium complex. The activation of eNOS also involves the mechanisms of phosphorylation and dephosphorylation. The phosphorylation state of eNOS plays a fundamental role in the regulation of its activity, so the phosphorylation of serial residues 1177, 633, and 615 leads to the activation of eNOS while the phosphorylation of residues serine 114 and threonin 495 inhibits eNOS [30].

##### Endothelium-Derived Hyperpolarizing Factor (EDHF)

EDHF hyperpolarizes the plasma membrane and relaxes the vascular smooth muscle cell through the putative potassium channels and insensitizes the cell to vasoconstrictor stimuli [31]. At the level of large vessels, endothelium-dependent relaxation is mainly related to NO, whereas at the microvessel, coronary, or peripheral levels, this relaxation is mainly dependent on EDHF [32]. In addition, in some arteries the effects of EDHF are totally dependent on the presence of gap junctions [33,34].

To date, the chemical nature of EDHF is still unknown, however several hypotheses have been made for H_2_O_2_ [35,36], potassium ions [37,38], epoxyeicosatrinoic acids (EETs are metabolites of arachidonic acid, synthesized by the endothelial cell under the action of cytochromes p450) [39,40], and nitroxyl (HNO) [41,42,43].

Under normal conditions, the production of NO regulates the production of contracting factors, and thus helps to keep the vessel in a dilated state. Under pathological conditions, vascular tone is altered by a decrease in the production of relaxing factors or the overproduction of contracting factors.

## 3. Vascular Dysfunction in Chronic Kidney Disease

**Figure 2:**
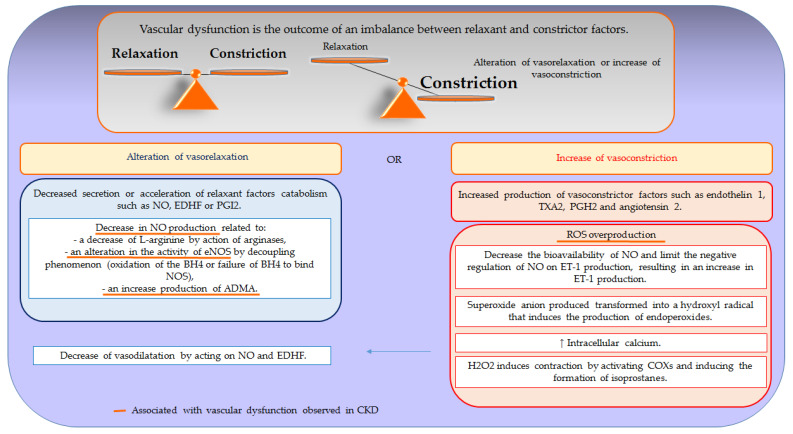
Mechanisms implicated in vascular dysfunction. NO, nitric oxide; EDHF, endothelium-derived hyperpolarizing factor; PGI2, prostacyclin; NOS, NO synthase; eNOS, endothelial NO synthase; BH4, tetrahydrobiopterin; ADMA, asymmetric dimethylarginine; ET-1, endothelin 1; TXA2, thromboxan A2; PGH2, prostaglandin H2; ROS: reactive oxygen species; COX, cyclooxygenase.

Figure 2 provides a schematic view of the mechanisms implicated in vascular dysfunction. 

Vascular dysfunction is the outcome of an imbalance between relaxing and contracting factors. This phenomenon is mostly due to a decrease in secretion or an acceleration of the catabolism of relaxant factors associated with or without an increased production of contracting factors, resulting in a reduction of vasorelaxation or a potentiation of vasoconstriction. Likewise, vascular dysfunction can also be related to a decrease in the sensitivity of VSMCs to factors produced by endothelium or an overproduction of ROS.

### 3.1. Nitric Oxide (NO)-Related Dysfunction

In many cardiovascular diseases, endothelial dysfunction is characterized by impaired relaxation associated with a decrease in the amount of NO combined or not with a loss of its effects.

The decrease in NO production can be mainly related to a decrease in the substrate of the enzyme (L-arginine) or an alteration in the activity of eNOS. The decrease in bioavailability of L-arginine is extremely rare given the large amounts of L-arginine produced by endothelial cells. It is mainly due to the action of arginases whose affinity for L-arginine increases in pathological situations [44,45]. The alteration of the activity of eNOS could be linked either to a decrease in the expression of this enzyme or to an increase in the expression of this same enzyme which becomes non-functional by a decoupling phenomenon. Different molecules are needed for the transformation of L-arginine into L-citruline by NOS. Among these molecules, tetrahydrobiopterin (BH4) plays an essential role. Indeed, the decoupling of the NOS can be associated with oxidation of the BH4 or a failure of BH4 to bind NOS [46,47]. The lack of NO production can also be related to an increase in the production of circulating ADMA, a natural analogue of L-arginine acting as an endogenous NOS inhibitor [48].

The loss of the NO effects can be caused, on the one hand, by the increase in its catabolism and, on the other hand, by the loss of the NO sensitivity of the guanylate cyclase present at the level of VSMCs [49].

### 3.2. The Role of Reactive Oxygen Species (ROS) in Vascular Dysfunction

Oxidative stress, linked to overproduction of ROS, induces a major deleterious effect in vascular function by reducing relaxation or potentiating contraction. 

ROS are able to reduce relaxation function by acting both on NO and EDHF. When they interact with NO, ROS form peroxynitrites (ONOO-) which decreases the bioavailability of NO and decreases the sensitivity and expression of guanylate cyclase, thus, contributing to a reduction in the effects of NO and the reduction of vasorelaxation [50,51]. In addition, excess ROS sets up a vicious circle leading to its continued production by inhibiting antioxidant systems. This results in a decoupling of eNOS which itself becomes a source of superoxide anion [47,52,53].

ROS and, in particular, superoxide anion decrease EDHF-induced vasorelaxation by decreasing the activity of calcium-sensitive potassium channels and acting on cellular junctions, and thus alter the spread of hyperpolarization of the endothelial cells at VSMCs [54,55].

ROS play a major effect in vascular contraction through different mechanisms. By decreasing the bioavailability of NO, ROS limit the negative regulation of NO on the production of ET-1, resulting in an increase in the production of ET-1, and therefore an increase in its contraction capacity. Another mechanism involves the superoxide anion which acts at the level of the VSMCs and induces the production of contracting prostanoids such as endoperoxydes and prostaglandins [56]. The hydroxyl radical also plays a role in inducing the contraction of the VSMCs through an increase in intracellular calcium [24].

In addition, H_2_O_2_ induces contraction by activating COXs, inducing the formation of isoprostanes which play a powerful vessel contraction role per action on TXA2 receptors [57,58,59]. ROS also play a direct role in the contraction of the VSMCs by influencing intracellular calcium homeostasis through the NADPH oxidases Nox 4 and 5. High levels of ROS cause a change in the RRya, alter the binding of actin proteins, and influence the TRPM2 calcium channel, resulting in calcium inflow and contraction.

### 3.3. Vascular Dysfunction in Chronic Kidney Disease

CKD is associated with higher cardiovascular mortality, and traditional risk factors do not fully explain this increase in risk. Vascular dysfunction is one of the characteristics of CKD and tends to emerge as a new, less classical risk factor. In CKD, vascular dysfunction can be demonstrated by the detection of circulating markers reflecting endothelium activation, such as von Willebrand factor, tissue factor, thrombomoduline, type I plasminogen inhibitor, intercellular adhesion molecule-1 (ICAM-1), vascular cells adhesion molecule-1 (VCAM-1), and P and E selectins [60,61,62]. Vascular dysfunction occurs early in the progression of CKD and appears independently of hypertension development. It has been observed, using a CKD animal model, that these vascular function changes are characterized by a defect in acetylcholine relaxation and by an overexpression of the ICAM-1 and VCAM1 adhesion molecules. In addition, these vascular alterations have been associated with the development of cardiac abnormalities and worsen during progression of CKD [63]. As well, endothelium dysfunction observed in CKD patients has also been reported to be the result of increased endothelial injury and decreased endothelial repair [64] and has been associated with numerous systemic alterations.

The vascular dysfunction observed in CKD patients has been associated with a decrease in NO production [9,10,65] mainly related to an effect on eNOS and increased production of ADMA [11,65]. In patients with CKD, increased oxidant activity associated with a reduced antioxidant capacity has been shown to be responsible for the considerable increase in oxidative stress [6,66]. These phenomena were largely due to the decrease in the activity of the SOD, the enzyme implicated in ROS detoxification, and to the increase in activity of enzymes responsible for ROS production such as NADPH oxidases and iNOS. Moreover, CKD has been characterized by systemic alterations such as inflammation [4] and the presence of uremic endothelium exhibiting a proinflammatory phenotype with an increased expression of adhesion molecules [63].

Endothelial microparticles (EMP) are vesicles of 0.1 to 1 µm in size, produced by plasma membrane shedding after cell activation or apoptosis and characterized by the externalization of phosphatidylserine and the presence of EC-specific surface antigens. An increase of EMP and circulating endothelial cells (CEC), observed in CKD patients [67,68], has been associated with vascular dysfunction [69] and, essentially, due to an inhibition of the NO pathway.

The vessels of CKD patients are permanently exposed to numerous uremic toxins that can be responsible for the observed vascular dysfunction.

## 4. The Role of Uremic Toxin in Vascular Dysfunction 

Table 1 summarizes the mechanisms by which uremic toxins impact vascular dysfunction.

In CKD, glomerular kidney filtration is less effective and numerous substances accumulating in the body are called uremic toxins UTs. The organic UTs can be classified according to their molecular weight and removal by dialysis into three classes according to the European Uremic Toxin Work Group (EUtox) [70,71] as follows:

1. Small water-soluble compounds with a maximum molecular weight (MW) of 500 Daltons (Da). The main molecules in this group include urea, creatinine, Pi, ADMA, and guanidine compounds, which are easily removed by dialysis.
2. Middle molecule compounds of moderately elevated MW (>500 Da). Many of these compounds are peptides, such as FGF-23 and PTH. They can only be removed by dialysis membranes with pores large enough to allow their passage.
3. Protein-bound compounds which are, generally, of low MW. The main molecules in this group are phenols, indoles (i.e., IS), and cresols (PCS). They are difficult to remove by dialysis.

The impact of uremic toxins on vessel function has been studied using serum samples from CKD patients. It was demonstrated that uremic toxins present in the serum of patients with CKD altered endothelial cell properties in vitro by reducing its proliferation and migration and inducing its apoptosis. Moreover, the uremic toxins altered the remodeling of the extracellular matrix in promoting the expression and activity of extracellular matrix-degrading proteinase such as MMP-2 and MMP-9 (matrix metallopeptidase 2 and 9), whereas they reduced the expression of the metallopeptidase inhibitors, TIMP-1 and TIMP-2 (tissues inhibitor of metalloproteinases 1 and 2) [72]. These studies using uremic serum have established the deleterious role of these toxins, but the composition of this uremic serum is very complex and novel toxins have been discovered regularly during the two past decades. Therefore, the implementation of studies using an isolated uremic toxin has been necessary to clarify the individual role of each of these toxins on vascular function.

A review of the vascular effects of several well-known uremic toxins, such as Pi, PCS, IS, FGF23, and other UTs which lead to UTs-induced vascular dysfunction, is given below.

### 4.1. Phosphate and Vascular Dysfunction

Inorganic phosphate is considered to be a uremic toxin since its accumulation leads to hyperphosphatemia which has adverse effects on many biological systems [73]. Pi is a uremic toxin that is part of the class of small water-soluble molecules with a molecular weight of less than 500 Da. Hyperphosphatemia is defined as phosphatemia greater than 1.5 mM. In the early stages of CKD, the gradual loss of functional nephrons leads to the accumulation of Pi which forms a complex with calcium leading, ultimately, to the formation of hydroxyapatite crystals. In order to prevent hyperphosphatemia, the body reacts via the activation of compensatory mechanisms, including an increase in FGF23 and PTH secretion. In advanced CKD, these mechanisms are unable to overcome the continuous input of Pi from dietary intake, leading to hyperphosphatemia.

Studies have shown that high phosphate levels are responsible for the development of vascular calcifications and, consequently, responsible for the development of cardiovascular diseases in CKD patients [74,75,76,77]. The deleterious effects of phosphate have also been observed in the absence of vascular calcifications, suggesting that another mechanism was responsible for the deleterious cardiovascular effects of phosphate and the idea of a direct role of phosphate on vascular function emerged. This role in vascular dysfunction was investigated, using an experimental mouse model with normal renal function or with CKD [78], and in healthy young male volunteers with intact renal function [79].

It was demonstrated that Pi exerts a rapid, concentration-dependent vasoconstriction effect on the aortas of sham-operated (i.e., non-CKD) mice. In the same manner, phosphate loading in vivo led to a dose dependent increase in phenylephrine-induced contraction and a decrease in endothelium-dependent relaxation of aortic rings ex vivo, independent of the presence or absence of CKD [78]. This study confirmed the results obtained by Shuto et al. who demonstrated that increased serum phosphate levels induced endothelial dysfunction in healthy volunteers [79].

Endothelium-dependent contraction is due either to reduced nitric oxide bioavailability or to the generation of endothelium-derived contracting factors such as oxygen-derived free radicals. The vasoconstriction effect of Pi has been found to be abolished by dimethylthiourea, a scavenger of hydroxyl radicals, confirming the role of oxidative stress in the vasoconstriction induced by phosphate. Using cell cultures, it was demonstrated that Pi increased the production of ROS in VSMCs and in endothelial cells via NADPH oxidase activation [78,80,81,82].

The decrease in endothelium-dependent relaxation is the result of a decrease in NO production [79,82] via an effect on the level of intracellular Ca^2+^ concentration that could inactivate eNOS [81] and an effect on eNOS phosphorylation on threonine 495 [78,79].

The induction of endothelial cells apoptosis and the loss of endothelial integrity can also explain the Pi effects observed on vascular reactivity [78,83]. The phosphate is able to induce apoptosis by increasing oxidative stress after 2 h of exposure and by disrupting the mitochondrial membrane potential and, subsequently, caspase activation.

All these effects are linked. The phosphate-induced apoptosis process is paired to an increase in oxidative stress. The oxidative stress is itself responsible for the decrease in NO production resulting in an increase in vascular contraction and a decrease in endothelium-dependent relaxation.

Moreover, the high phosphate levels induce EMP shedding which is responsible for a decrease of intracellular levels of annexin-II, and thus result in impaired endothelial cells with thrombotic, inflammatory, and anti-angiogenic properties [84].

Sevelamer is a calcium-free phosphate binder whose phosphate lowering effects are known to result in better control of serum PTH and FGF23 levels and to slow the progression of vascular calcification in CKD experimental models. Beyond its ability to control phosphate, sevelamer has pleiotropic effects such as correcting lipid abnormalities, reducing oxidative stress, and reducing markers of inflammation [85].

In animal models, it has been demonstrated that sevelamer is able to improve endothelial dysfunction ex vivo following phosphate exposure [78]. This effect on vascular function has been associated with an improvement in systolic expansion of the aortic root, pulse wave velocity, and diastolic function [86].

In a randomized controlled study, it was demonstrated that sevelamer treatment could improve endothelial dysfunction in nondiabetic CKD stage 4 patients, in association with improved flow-mediated dilation and increased serum fetuin-A levels [83].

### 4.2. Para-Cresyl Sulfate and Vascular Dysfunction

PCS is a uremic toxin of the protein-bound molecule class. Tyrosine is a non-essential aromatic amino acid produced from phenylalanine. Some of the unconsumed tyrosine is metabolized by commensal bacteria to form the para-cresol which is, then, metabolized in the intestinal lining by sulfotransferases and glucuronosyltransferases to give two metabolites, i.e., mainly PCS and less glucuronide para-cresyl [87,88,89]. In the plasma, 95% of PCS circulates bound to albumin in healthy subjects, as well as in CKD patients [90,91]. The average of PCS plasma concentration in healthy subjects varies between 14.9 µM (±9.0 µM) and 35.1 µM (±19.7 µM) [92,93]. However, this concentration significantly increases in CKD patients reaching 568.0 µM (±237.0 µM) quantified by LC-MS-MS, as PCS is mainly excreted by the kidneys via organic anion transporters OAT1 and OAT3 [94,95].

An elevated PCS plasma concentration (similar to a uremic plasma concentration) altered the vascular function and remodeling in vitro. Thus, PCS induced oxidative stress by increasing ROS production in human endothelial cells (HUVEC) and human VSMCs. This toxin also had an impact on vascular reactivity. Indeed, PCS increased phenylephrine-induced contraction in vessels by activating ROCK mediators. It also impaired vascular remodeling by inducing a decrease in the media and the lumen surface area of vessel. This remodeling is believed to be one of the causes of HTA seen in patients with CKD [96].

A study conducted in vivo, in the context of atherosclerosis, demonstrated that PCS administrated by oral gavage in Apo E knock-out mice enhanced leukocyte-endothelium adhesion by increasing adhesion molecule expression (ICAM-1 and VCAM-1) in the aorta. Moreover, an analysis of atherosclerosis formation in these aortas indicated that PCS decreased the collagen contents of atherosclerosis plaque, then, undermined plaque stability, which could explain the prevalence of atherosclerosis associated with CKD [97].

In a CKD rat group obtained by 5/6 nephrectomy, the effects of intraperitoneal injection of PCS (50 mg/kg/day) on endothelial barrier impairment and vascular leakage were studied. Vascular permeability changes were evaluated by the techniques of both Evans blue and India ink tracer and the aortic endothelial integrity was evaluated by increased immunoglobulin G leakage.

Increased endothelial leakage induced by PCS in skin microvessels and the aorta of CKD rats suggested that the PCS induced endothelial barrier dysfunction [98]. The endothelial dysfunction was also promoted by incubation of endothelial cells with increasing PCS concentration in vitro. Among the negative effects of PCS on endothelial function, a study noted the release of EMP by endothelial cells which are markers of endothelial dysfunction [99]. PCS was also implicated in the development of vascular calcification via stimulating the expression of osteoblastic markers such as alkaline phosphatase, osteopontin, and core-binding factor alpha1 in human aortic smooth muscle cells [100].

AST-120 (Kremezin^®^, Kureha Corporation, Tokyo, Japan) is an adsorbent composed of spherical carbon particles, administered orally, which can absorb various uremic toxins. Moreover, the oral administration of AST-120 significantly reduced PCS concentration in mice where renal failure was induced by adenine. Hence, these findings indicated that AST-120 changed the composition of gut microbiota in these mice via reducing the affluence of some bacteria implicated in the generation of para-cresol which is a precursor of PCS. Consequently, fecal para-cresol was low in the group treated by AST-120 as compared with the group not treated. In addition to this mechanism, AST-120 exerted its effect by adsorption of uremic toxin precursors such as para-cresol, and the AST-120 effect on vascular reactivity could be due in part to reduction of deleterious PCS effect [101].

Beta glucans found in whole grain cereals are fibers known for their ability to reduce low-density lipoprotein (LDL) and total cholesterol. Some results have shown their effects on the modulation of gut microbiota composition. A study analyzed the effects of beta glucan supplementation on PCS levels in healthy individuals who received a diet enriched with beta-glucans for a period of two months. The results showed that dietary treatment by beta glucans significantly decreased the levels of PCS and, as a consequence, increased markedly the flow mediated dilatation associated with endothelial function improvement [102].

### 4.3. Indoxyl Sulfate and Vascular Dysfunction

IS is a uremic toxin of the protein bound molecule class. Our diet is composed of proteins that are broken down into amino acids at the intestinal level. Some of these amino acids are absorbed in the intestinal lining and are found in the bloodstream, and other amino acids are degraded in the colon. Tryptophan undergoes this putrefaction process to give the indole. The formed indole is metabolized at the liver level to give IS.

In CKD, impaired absorption of amino acids leads to an increase during the rotting process and the gradual accumulation of IS. The continuous loss of functional nephrons leads to a decrease in the renal elimination of this compound and contributes to an increase in serum concentrations of this uremic toxin [103]. In patients with CKD, the IS level was a predictor of mortality [104,105]. The observational nature of the present studies prevented authors from demonstrating a causal relationship. In addition, the observed associations could be due to unknown or unmeasured residual confounders. The correlation between IS levels and cardiovascular events was controversial until the meta-analysis realized by Lin et al. In this analysis, they included five studies which differed in terms of follow up of patients and dialysis technics performed [106]. The authors found no association between IS plasma levels and cardiovascular outcomes. This conclusion was moderated by potential confounders between IS plasma levels and nutritional intake, as long as reduced protein intake negatively impacted IS plasma levels. In some observational studies, plasma levels of free and not total protein-bound UTs were associated with hard outcomes. Vanholder et al. performed a systematic review, selecting only studies in which, depending on the albumin concentration, real or extrapolated free concentrations of indoxyl sulfate and para-cresyl sulfate remained in the uremic range. To conduct this review, a quality score was developed to allow the retrieval of methodologically correct studies unbiased by erroneous conditions related to albumin binding. Their data confirmed the toxicity of IS and PCS in vascular disease progression [107].

Furthermore, a novel study investigated the association between serum IS levels and endothelial function in patients with stages 3–5 CKD. The endothelial function, represented by the vascular reactivity index (VRI), was measured non-invasively using digital thermal monitoring and IS level was associated inversely with the VRI values demonstrating a modulating role of IS in endothelial function [108]. In future papers it would be interesting to analyze the effect on vascular dysfunction taking into account albumin and IS parameters or total and free IS levels at each CKD stage.

IS plays a deleterious role on vascular function in many ways. IS reduces endothelium-dependent relaxation via a decrease in NO production and is able to reduce the viability of endothelial cells and induce ROS production via an activation of NADPH oxidase activity and the inhibition of antioxidant systems [109,110,111,112,113,114,115].

Recently, Matsumoto et al. detailed the mechanisms implicated in the alteration of vascular dysfunction induced by IS [116]. They measured acetylcholine (ACh)-induced endothelium-dependent relaxation in the absence and presence of several inhibitors. The reported that only under the inhibition of NOS, was the difference of sensitivity to ACh between vehicle and IS eliminated. These findings indicated that acute exposure of IS impaired NO signaling but not EDHF or vasodilator prostaglandins.

IS has also been shown to be able to induce the expression of adhesion molecules such as E selectin, ICAM-1, and monocyte chemotactic protein-1 (MCP-1) [111,117,118]. Moreover, the action of IS on cell junction participated in the deleterious effect on endothelial integrity [114,115,116,117,118,119]. IS also acted on the number of the endothelial cells, and therefore promoted endothelial progenitor cell senescence [120,121].

AST-120 inhibits the liver synthesis of IS by blocking the gastrointestinal absorption of its biochemical precursor indole. Several studies have looked at the effects of AST-120 on vascular function or vascular rigidity. AST-120 was able to restore vascular function in an experimental model of CKD associated or not with atherosclerosis in improving NO bioavailability [109].

It has been demonstrated that AST-120 decreased carotid intima-media thickness and arterial stiffness in non-dialysis patients with chronic renal failure [122] and improved vascular function in humans [114]. Further studies were subsequently conducted and one of them looked at the effects of AST-120 on the microvascular function. Fourteen patients were treated with AST-120 (6 g/day) for six months. An improvement of the endothelium-dependent microvascular function was induced by this treatment [123].

### 4.4. Klotho and FGF23 and Vascular Dysfunction

The FGF23 is a bone-derived hormone that controls mineral homeostasis. In the kidney, FGF23 reduces the circulating level of phosphate by increasing its excretion and 1.25-(OH)_2_-vitamin D_3_. In the parathyroid gland, FGF23 also inhibits the secretion of PTH [124,125,126,127,128]. In these two organs, FGF23 binds and activates the FGF-receptor in combination with the co-receptor klotho [129].

Klotho was initially discovered as an anti-aging factor because genetically modified mice lacking klotho presented a premature aging phenotype [130]. It is known that the circulating klotho level declines with advancing age [131]. Klotho is mainly expressed in the kidney but is also detectable in other organs such as parathyroid glands, choroid plexus, and human vascular tissue [132,133]. Klotho is known both as a membrane protein and as a soluble form. The soluble form of klotho could be generated either through an alternative splicing and directly released into extracellular compartment, from renal tubule and probably from extra-renal organs [134,135,136], or by a cleavage of the membrane klotho extracellular domain [130,133,137,138]. Additionally, genetic variants, which seem to have relevance with aging, can affect its secretion. Notably, homozygous expression of the KL-VERSUS variant has resulted in a lower secretion of klotho as compared with the heterozygous expression of this klotho variant, leading to cardiovascular disease [139,140,141,142]. In addition to its role as a co-receptor, klotho is also a factor which exerts an anti-aging effect by suppressing growth factor signaling and oxidative stress, and, in particular, regulating the activity of several ion channels without FGF23 [143,144].

FGF23 and klotho signaling plays an important role in heart and blood vessels functions and structures [145,146,147,148]. *Klotho*^-/-^ and *fgf23*^-/-^ deficient mice developed identical phenotypes, characterized by accelerated aging, a severe hypercalcemia and hyperphosphatemia, and subsequent multiple organs dysfunction such as atherosclerosis, ectopic calcifications, bone demineralization, skin atrophy, and emphysema [125,130,149]. Similar to the mice model, the loss-of-function mutations in KLOTHO or FGF23 in humans conduces to a pathological serum level of active vitamin D, calcium, phosphate, and the observation of an ectopic calcification such as vascular calcification [147,150]. A transgenic overexpression of klotho has been shown to extend the life span in mice, promote heart protection, and reduce oxidative stress [143,144,151].

Many studies have suggested that klotho and FGF23 play a role in vascular dysfunction. A deregulation of the klotho/FGF23 state observed in CKD has also been linked with cardiovascular disease such as left ventricular hypertrophy, vascular calcification, and endothelial dysfunction [152,153,154,155,156,157]. Klotho deficiency followed by a high serum level of FGF23 was observed [154]. However, their combined effects on the vessel wall seemed to be complex with opposing actions in terms of local oxidant generation and NO production [158].

CKD is considered to be a condition of klotho deficiency. A lower level of circulating klotho can induce arterial stiffness and hypertension via a downregulation of SIRT1 expression in endothelial and smooth muscle cells. These effects have been associated with a decrease in AMP-activated protein kinase activity and eNOS expression, and they contributed to age-related arterial stiffening induced by klotho deficiency [159]. Moreover, klotho deficiency upregulated NADPH oxidase activity and superoxide production, increased collagen expression, enhanced elastin fragmentation in the media of aortas, and also increased MMP-2 and MMP-9 expression [160]. Klotho deficiency also impaired aortic endothelial function and its subsequent vasorelaxation [161]. In contrast, *Klotho* gene delivery improved vascular function by increasing endothelial-derived NO production and reducing oxidative stress in VSMCs, resulting in prevention of medial hypertrophy in the aorta [162,163].

Independent of FGF23, klotho directly has an effect on vascular function where it plays a dual role. On the one hand, klotho is able to stimulate the production of NO, thus, mitigating the contraction induced by FGF23 and causing the relaxation of vessels constricted by phosphate. On the other hand, klotho is able to drive the production of ROS when used at a high concentration which mitigates its beneficial effect [158]. Klotho modulates the stress response in human senescent endothelial cells [164]. More precisely, the increased ROS degradation by klotho results from the activation of the Foxo forkhead transcription factor 3a (Foxo3a) that is negatively regulated by insulin/IGF1 signaling, thereby, inducing expression of SOD2 and CAT. In the presence of klotho, FGF23 induced NO release in human coronary artery endothelial cells and its stimulating effects on ROS production were counterbalanced by increased ROS degradation (via a Foxo3a mediated activation of antioxidants, SOD2, and CAT) [165]. Finally, although all these data show that klotho deficiency is linked to cardiovascular risk, some clinical studies with CKD patients have shown that soluble klotho is independently associated with arterial stiffness and did not predict atherosclerotic or acute heart failure events [166].

Klotho acts as a mandatory co-receptor of FGF 23 in various tissues but FGF 23 exerts equally an effect independent of klotho [167]. Numerous studies have shown that a high circulating level of FGF23 was linked to vascular dysfunction and cardiovascular mortality [145,146,147,148,168,169]. FGF23 has a deleterious effect on vascular function by increasing the production of ROS. The deleterious effect of FGF23 can be associated with an increase of vasoconstriction [158], or with impaired vasorelaxation [146]. Previous studies have observed that FGF23 induced an increase in oxidative stress, reduced NO production, induced the expression of cell adhesion molecules, and abolished endothelium-dependent relaxation [165,170,171]. High levels of FGF23 have been shown to be clinically associated with endothelial dysfunction and arterial stiffness [155]. In a non-CKD population, higher levels of FGF23 were independently associated with an increased risk of major cardiovascular events [172] but could be associated with carotid atherosclerosis [173].

The impact of elevated FGF23 on endothelial function was studied ex vivo using animal models. However, the difference of FGF23 concentration and vessel types used complicated the interpretation of the direct vascular response to FGF23. Some studies failed to observe the effects of FGF23 in aortic rings and mesenteric arteries [174], whereas another study found that FGF23 directly caused contraction of the mouse aortic vessels via an effect on ROS production [158]. To further confirm the latter results, in states of klotho deficiency (CKD), FGF23-mediated NO synthesis was blunted and ROS formation overruled ROS degradation. Thus, FGF23 excess primarily promoted oxidative stress, and thus endothelial dysfunction [165,175].

### 4.5. Other Uremic Toxins: ADMA, SDMA, AGEs, Urea and Vascular Dysfunction

One of the earliest occurrences of endothelial dysfunction was due to NO deficiency. The dimethylarginines ADMA and symmetric dimethylarginine (SDMA), its structural isomer, are metabolites of L-arginine, which is the amino acid catalyzed to L-citrulline and NO by NOS. ADMA and SDMA have been listed as uremic toxins among the guanidine compounds and are considered as small, water-soluble molecules.

Both ADMA and SDMA are implicated in different pathologies and the changes in their expression levels have different consequences. ADMA appears as a marker of cardiovascular disease, whereas SDMA identifies reduced renal function.

ADMA is a competitive inhibitor of NOS, thereby reducing NO production and promoting endothelial dysfunction. An elevated ADMA level has been associated with an increased risk for mortality and cardiovascular events in a general population and in CKD patients [11,176,177]. ADMA acts on endothelial cells by accelerating the senescence and inducing oxidative stress [178]. Moreover, in a general population, elevated plasma ADMA concentrations have been associated with decreased brachial flow mediated relaxation responses in healthy adults [179] and ADMA was targeted as a new biomarker for endothelial dysfunction [48]. ADMA acts on vascular dysfunction by inhibiting the synthesis of the endothelial-derived relaxing factors, such as NO [11].

SDMA interferes indirectly with NO production by reducing the cellular availability of arginine. In the same manner, it has been demonstrated that SDMA exerts an inhibitory effect on NO production [180]. Additionally, SDMA induced ROS production via NADPH oxidase activation through endothelial Toll-like receptor 2 [181].

Advanced glycation end products (AGEs) constitute a heterogeneous group of compounds derived from the nonenzymatic glycation of proteins, lipids, and nuclear acids through a complex sequence of reactions referred to as the Maillard reaction [182]. They accumulate in patients with CKD, are classified as uremic toxins, and are considered to be biomarkers. AGEs are sequestrated via binding to advanced glycation end products receptor 1 (AGER1) resulting in reduction of AGEs levels and reduction of oxidative stress [183]. Another receptor, advanced glycation end products-specific receptor (RAGE), is able to bind many ligands, including AGEs [184]. An increase of RAGE expression has been demonstrated in CKD [185]. The most important effect of AGEs on CKD is devoted to SMCs where AGEs play a role in osteogenic-like differentiation of SMCs and subsequent calcification [186,187]. Moreover, the interaction between AGEs and RAGE induced the increase of ROS production resulting in an increase of SMC proliferation in vitro [188]. Focusing on endothelial cells, AGEs induce vascular dysfunction in many ways as follows: AGEs promote inflammation and oxidative stress [189] via activation of NADPH oxidase [190], induce an upregulation of adhesion molecules expression [188], and play an important role in endothelial dysfunction by downregulation of NOS and, accordingly, reduction of NO production [190,191,192].

AGEs are also able to induce vascular contraction by modulating ET-1 expression. The ET-1 transcription is regulated by nuclear factor-kappaB in AGEs-stimulated cultured endothelial cells [193]. AGEs act directly on endothelial cells by inducing endothelial cells apoptosis and impairing endothelial progenitor cells survival, differentiation, migration, and function [194,195,196].

Urea has emerged recently as having a possible direct and indirect toxicity [197,198]. Urea is responsible of the carbamylation process in which charge of proteins are changed with consequences in modification proteins structure, function and consecutively interactions. [197]. Urea has a direct effect on VSMCs in inducing the calcification of aortic rings from CKD rats with urea enriched diet and cultures VSMC [199]. Moreover, the exposure to urea has been associated with upregulation of VSMCs apoptosis by upregulation of BAD, the agonist of intrinsic apoptosis pathway [200].

Concerning endothelial cells, D’Apolito et al. found that urea, at the concentrations seen in CKD patients, increased ROS production directly in arterial endothelial cells. These effects were associated with increased expression of VCAM-1, MCP-1, accumulation of AGEs as well as inhibition of glyceraldehyde- 3-phosphate dehydrogenase (GADPH) and increase of endothelial progenitor cells senescence [201,202]. More recently, the authors show that transient exposure to urea induces ROS production in human aortic endothelial cells that persists for days after urea is removed, suggesting a cellular memory for urea-induced oxidative stress in endothelial cells [203].

## 5. Conclusions

In patients with CKD, vascular dysfunction is a new risk factor. Uremic toxins present in the serum of patients with CKD alter endothelial cell properties, but the composition of this uremic serum is very complex. Each toxin can play its own role on vascular dysfunction and there are three therapeutic options to reduce endothelial dysfunction induced by uremic toxins. The first option could be to reduce uremic toxin concentrations using new dialysis modalities, the second option could consist of using drugs such as AST-120 or Sevelamer to quench uremic toxins, and the last option could be to counter uremic toxin effects by using anti-oxidants or by acting on the NO pathway.

## Figures and Tables

**Table 1:** The mechanisms by which uremic toxins impact vascular dysfunction.

Uremic Toxins	Effect on Vascular Reactivity
Phosphate	Vasoconstriction and decrease of vasorelaxation, decrease in NO production, stimulation of ROS production, induction of endothelial cells apoptosis.
p-cresyl sulfate	Vasoconstriction, stimulation of ROS production, increase in EMP release, vascular remodeling.
Indoxyl sulfate	Decrease of endothelium dependent vasorelaxation, decrease of NO production, stimulation of ROS production, reduction of endothelial cells viability.
Klotho deficiency	Arterial stiffness, decrease of eNOS expression, stimulation of ROS production, decrease of vasorelaxation.
FGF23	Stimulation of ROS production, increase in vasoconstriction or decrease of vasorelaxation, reduction of NO production.
ADMA, SDMA	Inhibition of NO synthase, stimulation of ROS production.
AGE	Stimulation of ROS production, inhibition of NO synthase activity, induction of ET-1 expression.
